# *In Vitro* Biofilm Formation on Aryl Ketone Polymer (AKP), A New Denture Material, Compared with That on Three Traditional Dental Denture Materials

**DOI:** 10.1155/2021/4713510

**Published:** 2021-10-26

**Authors:** Cadie Martin, Laura Purevdorj-Gage, Wei Li, Timothy J. Shary, Bin Yang, Ryan J. Murphy, Christine D. Wu

**Affiliations:** ^1^Microbiology and Environmental Research & Innovation Center–Bristol, Solvay, 350 George Patterson Blvd., Bristol, PA 19007, USA; ^2^Department of Pediatric Dentistry, University of Illinois at Chicago, College of Dentistry, 801 S. Paulina Street, Chicago, IL 60612, USA; ^3^Solvay Specialty Polymers, 4500 McGinnis Ferry Road, Alpharetta, GA 30005, USA; ^4^Department of Restorative Dentistry, University of Illinois at Chicago, College of Dentistry, 801 S. Paulina Street, Chicago, IL 60612, USA

## Abstract

Control of denture plaque biofilms is a practical approach to preventing persistent oral infections such as denture stomatitis. *Objectives*. This study compared *in vitro* biofilm attachment and growth on a new denture material, Ultaire® AKP, with that on traditional denture materials including cobalt chrome (CoCr), polymethyl methacrylate (PMMA), and polyoxymethylene (POM). *Methods*. Microbial biofilms were grown with cultures of *Candida albicans*, *Streptococcus mutans* UA159, or a mixed *Streptococcus* spp. (*S. mutans* 700610/*Streptococcus sanguinis* BAA-1455) for 6 hours in a static protocol or 24 hours in a dynamic protocol for each material. Adherent biofilm cells were removed, and viable colony-forming units (CFUs) were enumerated. Confocal microscopy of the 24-hour *Streptococcus* spp. biofilms was used to determine biofilm mass and roughness coefficients. *Results*. The rank order of *C. albicans* attachment after 6 hours was CoCr > PMMA^*∗*^ > Ultaire® AKP^*∗*^ (^*∗*^vs CoCr, *p* ≤ 0.05), and that for 24-hour biofilm growth was CoCr > Ultaire® AKP^*∗*^ > PMMA^*∗*^ (^*∗*^vs CoCr, *p* ≤ 0.05). The rank order of *S. mutans* biofilm attachment was CoCr > POM > Ultaire® AKP^*∗*^ > PMMA^*∗*^ (^*∗*^vs CoCr, *p* ≤ 0.05), and that for the 24-hour *Streptococcus* spp. biofilm growth was POM > Ultaire® AKP > PMMA > CoCr^*∗*^ (^*∗*^vs POM, *p* ≤ 0.05). Confocal images revealed structural differences in *Streptococcus* spp. biofilms on CoCr compared with the other test materials. Significantly lower roughness coefficients of *Streptococcus* spp. biofilms on Ultaire® AKP were noted, suggesting that these biofilms were less differentiated. Ultaire® AKP promoted significantly less *C. albicans* and *S. mutans* biofilm attachment than CoCr at 6 hours and *C. albicans* growth at 24 hours. *Streptococcus* spp. biofilms on Ultaire® AKP were less differentiated than those on other test materials. *Conclusion*. In addition to its material strength, Ultaire® AKP represents an attractive option for denture material in removable partial dentures.

## 1. Introduction

In developed countries, analysis of the epidemiological data on edentulism levels [1], as well as the social [2, 3] and economic [4] impact of tooth loss, suggests a global need for custom-manufactured removable partial dentures (RPDs). The materials used in RPD construction are ideally nontoxic, nonirritating, resistant to abrasion, and able to withstand repeated masticatory forces through excellent mechanical strength, resilience, and elastic properties [5]. These include metal (cobalt chrome (CoCr)), acrylic resin polymers (polymethyl methacrylate (PMMA)), and acetal resin polymers (polyoxymethylene (POM)). CoCr is a material with high strength and stiffness, but, due to its metallic nature, it has generally poor esthetics and may cause oral galvanism, osteolysis of abutment teeth, and inflammation [5]. Although polymers are esthetically superior to metal, common RPD polymers can weaken in wet environments [6], cause cytotoxicity due to monomer leaching [5], and develop surface abrasion after being cleansed [7]. Current acrylic polymer materials are not recommended for use alone as long-term RPD frameworks [8], because they lack strength and rigidity and tend to fracture due to impact or fatigue failure [7].

In addition to mechanical properties, the ability of an RPD material to resist oral biofilm attachment and colonization is important. For the denture-wearing population, microbial biofilm growth on dentures has the following effects: affecting gingival health, risk of caries, enamel demineralization [9, 10], risk of oral inflammation in the form of denture stomatitis [11], and risk of oral-inflammation-associated systemic illnesses such as cardiovascular disease [12], endothelial dysfunction [13], and aspiration pneumonia [14]. Treatment includes antifungal medication [15], denture disinfection, and continued good oral hygiene [16]. However, relapse often occurs after treatment ends [10, 16].

Difficulties in the treatment of biofilm-related diseases may be due to microorganism resilience within the structure of the biofilm [17]. Dental plaque biofilm is a complex, multicomponent microecosystem composed of mixed-microorganism communities on nonshedding surfaces within the mouth [18]. Plaque biofilms can colonize removable partial dentures (RPDs), and *Candida* spp., *Streptococci,* and opportunistic oral pathogens have frequently been detected [10, 11, 19, 20].

The surface characteristics of denture materials, such as roughness, charge, and hydrophobicity have been reported to affect bacterial adhesion and biofilm development [9, 18]. The development or modification of denture materials to resist biofilm growth would reduce the need for treatment of oral inflammation and diseases caused by denture-related biofilm [20].

Ultaire® AKP is a new high-performance aryl ketone polymer (AKP) that has unique mechanical properties. Ultaire® AKP demonstrates elasticity and flexural strength superior to those of current RPD polymer materials (e.g., PMMA and POM) and is similar to CoCr in its resistance to water sorption and solubility, high impact strength, and heat resistance (data on file; Solvay Dental 360®). In addition, Ultaire® AKP resists cleaner-induced surface roughness better than polymers (data on file; Solvay Dental 360®), and Ultaire® AKP outperformed CoCr in a clasp fatigue test [21]. Examples of how Ultaire® AKP may improve the RPD framework fitting through computer-aided design have been previously published [5]. However, oral microbial biofilm formation on Ultaire® AKP has not been examined.

The present *in vitro* study aimed to evaluate attachment and biofilm growth on Ultaire® AKP by oral microorganisms and compare it with those on three traditional denture materials: cobalt chrome (CoCr), acrylic resin (PMMA), and acetal resin (POM). The microorganisms used in this study were *Candida albicans*, *Streptococcus mutans*, and *Streptococcus sanguinis*. Biofilm attachment and growth were evaluated by the standardized biofilm assays, and the COMSTAT image analysis program was used to quantify and statistically compare the 3-dimensional attributes of the biofilms.

## 2. Materials and Methods

### 2.1. Materials

The four test materials used were the acrylic resin cobalt-chromium (CoCr, United Performance Metals, South Windsor, CT); polymethyl methacrylate (PMMA); the acetal resin polyoxymethylene (POM) (all purchased from Dental Art Laboratory Inc., Peoria, IL); and Ultaire® AKP (Solvay Specialty Polymers, Alpharetta, GA).

### 2.2. Biofilm Attachment Assay (6-Hour Static Method)

The test materials used were PMMA, CoCr, and Ultaire® AKP. Test materials were cut into coupons (1.2 cm × 0.7 cm × 2 mm), polished with decreasing coarseness of water-resistant silicon carbide abrasive paper to comparable surface roughness (Microcut grit P800 to P4000; Buehler, Lake Bluff, IL), and used for attachment assays [22]. The test microorganisms used for single-species biofilms were *S. mutans* UA159 grown in Brain Heart Infusion Broth and *C. albicans* ATCC 90028 grown in Sabouraud Dextrose Broth (both purchased from Becton, Dickinson and Co., Sparks, MD). Coupons were precoated with artificial saliva (100 mL, composed of 1.0 g Lab Lemco, 5.0 g proteose peptone, 2.0 g yeast extract, 0.35 g NaCl, 0.2 g CaCl_2_, 0.2 g KCl, 2.5 g Mucin type III, and 1.3 mL of 40% *w*/*v* urea) for 30 minutes at 37°C and placed in multiwell culture plates containing respective growth media. The wells were inoculated with either *C. albicans* (1 × 10^6^ CFU/mL) or *S. mutans* (1 × 10^6^ CFU/mL), and all plates were incubated at 37°C for 6 hours. *S. mutans* were incubated anaerobically (Forma Scientific Anaerobic System; 5% H_2_, 5% CO_2_, 90% N_2_). Control wells contained test organisms in growth media without test dental materials. After the attachment period, the coupons were gently rinsed three times with PBS and the attached cells were ultrasonically removed from coupon surfaces, serially diluted, and plated onto respective growth medium agar. The viable colonies were enumerated after incubation at 37°C for 24–48 hours. Duplicate independent experiments with a total of 6 replicate coupons were performed for each test material (*n* = 6).

### 2.3. Biofilm Growth Assay (24-Hour Dynamic Method)

For this assay, test materials used were PMMA, CoCr, Ultaire® AKP, and POM (a commonly used industry benchmark material in RPD construction). The coupon size prepared had 1.27 cm diameter × 3 mm thickness according to the protocol adapted from the ASTM protocol E2562-17 (http://www.astm.org) for the Centers for Disease Control and Prevention (CDC) Biofilm Reactor (BioSurface Technologies Corp.). The test organisms used were *C. albicans* ATCC 10231 or a dual-species biofilm containing *S. mutans* ATCC 700610 (75%) and *Streptococcus sanguinis* ATCC BAA-1455 (25%). This ratio was selected to mimic the *S. mutans/S. sanguinis* ratio described in a human caries-free oral environment [23]. The CDC Biofilm Reactor combines the elements of a continuously stirred tank reactor with a continuous flow reactor to provide an *in vitro* growth environment that controls the variables of shear, flow, and nutrient supply in microorganism adhesion and biofilm development [24]. The resulting biofilms are often thick and reproducible in multiple settings, and large quantities of mature biofilms can be produced in a short period of time [25, 26]. The CDC Biofilm Reactor is the only intralaboratory validated instrument for biofilm growth approved by both the CDC and the Environmental Protection Agency for its scientific rigor and reproducibility [24].

Vertically oriented round coupons prepared from test dental materials were precoated with artificial saliva [27] for 30 minutes at 40°C. The coupons were then spun on a baffle (125 rpm) for 6 hours at room temperature (23°C) in 3 g/L Tryptic Soy Broth with 0.5% sucrose for *C. albicans* (aerobically) and with 0.1% sucrose for *Streptococcus* spp. mix (anaerobically) with nitrogen. The attached cultures were then grown for another 18 hours with continued baffle stirring and aerobic media flow at a rate of 6.7 mL/min to discourage planktonic growth within the reactor vessel. After a total of 24 hours, coupons were removed and washed in sterile Butterfield's buffer solution (Hardy Diagnostics D599) and the attached biofilm cells were scraped with a sterile cotton swab for 1.5 minutes on each side of the coupon. Scraped disks and swabs were placed in vials containing 10 mL PBS, vortexed, and sonicated for 5 minutes at 40 kHz to dislodge the biofilm cells. The cell suspensions were serially diluted to 1 : 10,000 with sterile Butterfield's buffer solution and plated on potato dextrose agar for *C. albicans* or on BHI agar for the *Streptococcus* spp. mix. The viable counts (CFUs/coupon) were enumerated after incubation for 24–48 hours at 25°C for *C. albicans* or at 37°C in anaerobic jars (BD GasPak EZ Container System) for *Streptococcus* spp. Since the CDC Biofilm Reactor is designed to hold a maximum of 24 coupons for each experiment, the sample size for each of the 4 test materials was *n* = 6. For *C. albicans*, 5 replicates of each test material were used due to limited supply at the time (*n* = 5).

### 2.4. Confocal Microscopy

After 24 hours of *Streptococcus* spp. biofilm growth, disks were removed from the CDC Biofilm Reactor, washed, and stained with the LIVE/DEAD BacLight Bacterial Viability Kit (ThermoFisher Scientific: L7012) as per manufacturer's instructions. The *Streptococcus* spp. biofilms were imaged with a Leica TCS SP8 inverted confocal microscope. Images were analyzed for the measurement of biomass thicknesses and roughness coefficients with the COMSTAT analysis extension [28] through ImageJ software (https://imagej.nih.gov/ij/download.html). Each confocal image was composed of two channels, both of which were included in the COMSTAT analysis. COMSTAT data analysis included 7–10 coupon replicates per test material.

### 2.5. Statistical Analysis

To evaluate the bacterial attachment and biofilm growth on test materials, the viable bacterial CFUs attached to each material were compared to each other material separately. An unpaired, two-tailed Student's *t-*test was used for statistical comparisons of all sample means, with significance appointed at *p* ≤ 0.05. All data were analyzed with Minitab 17 statistical software.

## 3. Results

As shown in Table 1, the highest amount of biofilm attachment and growth for *C. albicans* were on CoCr. When compared with CoCr, Ultaire® AKP demonstrated up to 85% and 48% reduction in attachment and growth, respectively. When compared to materials other than CoCr, Ultaire® AKP promoted comparable biofilm growth of *C. albicans*. For *S. mutans*, the highest amount of attachment was also observed on CoCr. A 77% reduction in attachment was observed in Ultaire® AKP, when compared to CoCr. For 24-hour *Streptococcus* spp. biofilm growth, POM promoted the highest amount of biofilm. A 62% reduction was noted on Ultaire® AKP compared to POM.

**Figure 1:**
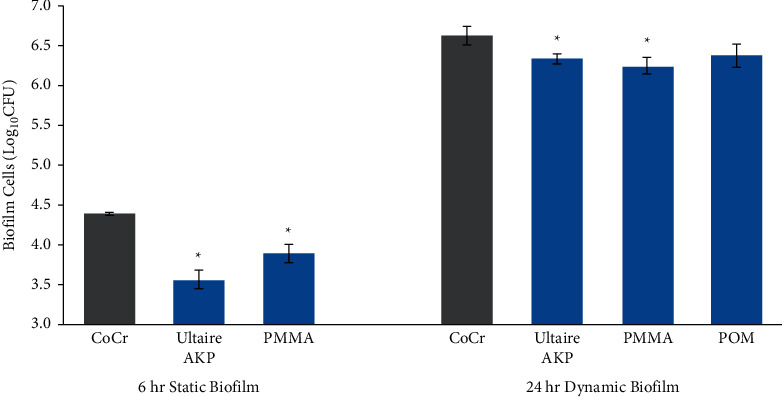
*Candida albicans* biofilms grown on Ultaire® AKP and traditional dental materials quantified as colony-forming units (CFUs) recovered from coupons after incubation with *C. albicans* for 6 hours under static conditions or for 24 hours under dynamic conditions in the CDC Biofilm Reactor. Solid bars represent mean log_10_ CFU/coupon, and error bars represent the standard error. Gray bars are materials with highest number of CFUs. ^*∗*^*p* ≤ 0.05 vs gray bar. An unpaired, two-tailed Student's *t-*test was used for statistical comparisons of all sample means, with significance appointed at *p* ≤ 0.05.

**Figure 2:**
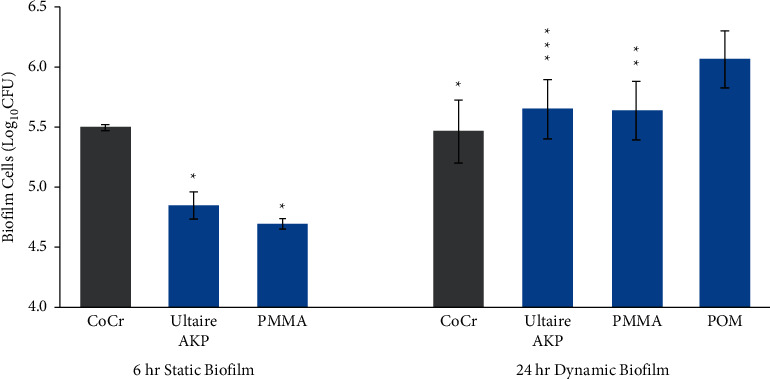
*Streptococcus mutans and Streptococcus* spp. biofilms grown on Ultaire® AKP and traditional dental materials, quantified as colony-forming units (CFUs) recovered from coupons after incubation with *S. mutans* for 6 hours under static conditions or a mix of *S. sanguinis* (75%) and *S. mutans* (25%) for 24 hours under dynamic conditions. Solid bars represent mean log_10_ CFU/coupon, and error bars represent standard error. Gray bars are materials with the highest number of CFUs. ^*∗*^*p* < 0.05 vs gray bar; ^*∗∗*^*p*=0.10 vs gray bar; ^*∗∗∗*^*p*=0.101 vs gray bar.

Compared with CoCr, significant reductions in *C. albicans* attachment and growth were observed on both Ultaire® AKP (*p* ≤ 0.05, Figure 1) and PMMA (*p* ≤ 0.05, Figure 1). Similarly, the attachment by S*. mutans* on Ultaire® AKP and PMMA were significantly reduced compared to CoCr (*p* ≤ 0.05, Figure 2). As already noted, *Streptococcus* spp. biofilm growth was highest on POM, and the reduced growth with other materials were at different significance levels: CoCr (*p* ≤ 0.05), Ultaire® AKP (*p*=0.101), and PMMA (*p*=0.100) (Figure 2).

**Figure 3:**
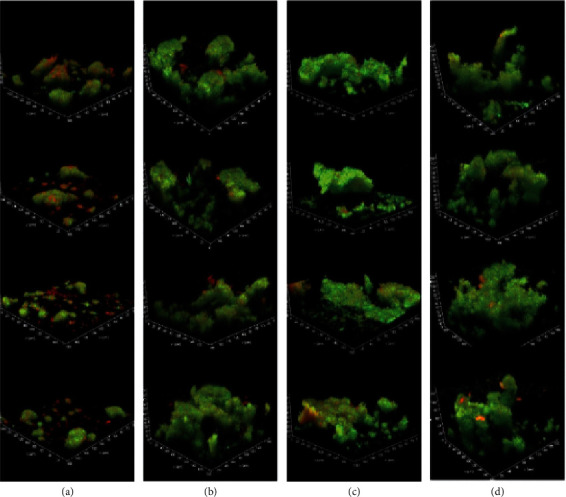
Three-dimensional confocal microscopy images (600x) from *Streptococcus* spp. biofilms grown on (a) CoCr, (b) Ultaire® AKP, (c) PMMA, and (d) POM for 24 hours under dynamic conditions in the CDC Biofilm Reactor. Each panel shows multiple images from a single representative biofilm.

**Figure 4:**
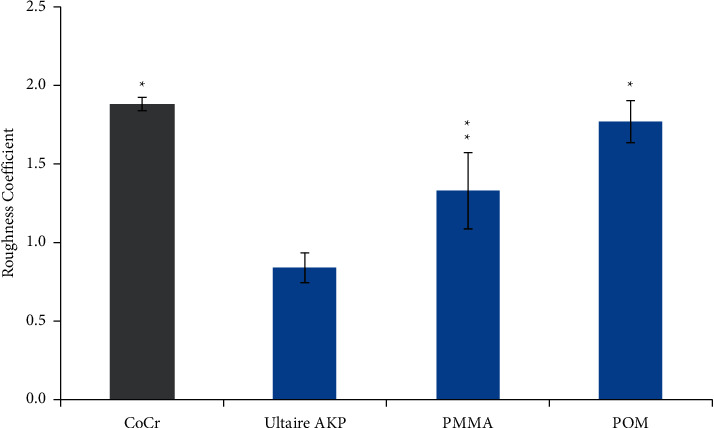
Roughness coefficients of *Streptococcus* spp. biofilms (average ± SE) grown on Ultaire® AKP and traditional dental materials for 24 hours under dynamic conditions in the CDC Biofilm Reactor. Gray bar is the highest biofilm roughness coefficient. ^*∗*^*p* ≤ 0.01 vs Ultaire® AKP; ^*∗∗*^*p*=0.02 vs Ultaire® AKP.

For the examination of structural differences in 24-hour *Streptococcus* spp. biofilm growth among the test materials, confocal microscopy was used to quantify the 3-dimensional architecture of the biofilms (Figures 3 and 4). The *Streptococcus* spp. biofilms on CoCr were sparse and distributed in a dense, island-like pattern, while the biofilms observed on Ultaire® AKP, PMMA, and POM polymers were larger, with more pronounced mushroom-like structures (Figure 3).

The mean *Streptococcus* spp. biofilm thicknesses as measured from the confocal image stacks were CoCr, 2.18 ± 2.32 *μ*m^3^/*μ*m^2^; Ultaire® AKP, 27.65 ± 8.26 *μ*m^3^/*μ*m^2^; PMMA, 20.03 ± 21.16 *μ*m^3^/*μ*m^2^; and POM, 6.96 ± 13.12 *μ*m^3^/*μ*m^2^. The thickness on Ultaire® AKP was significantly greater than on CoCr (*p* ≤ 0.01) and on POM (*p* ≤ 0.01). However, the mean *Streptococcus* spp. biofilm roughness coefficient was the lowest on Ultaire® AKP (0.84 ± 0.25) and was significantly different from that on CoCr (1.88 ± 0.13; *p* ≤ 0.01), PMMA (1.33 ± 0.69; *p*=0.02), and POM (1.77 ± 0.35; *p* ≤ 0.01) (Figure 4).

## 4. Discussion

The methodology established in this investigation allowed for study of the *in vitro* attachment and subsequent biofilm growth of *C. albicans* and cariogenic *Streptococci* on selected dental materials. Significantly less *C. albicans* attachment and biofilm growth were observed on Ultaire® AKP and PMMA polymers than on CoCr. However, significantly less *S. mutans* attachment was found on Ultaire® AKP and PMMA polymers than on CoCr, and *Streptococcus* spp. growth on Ultaire® AKP was not significantly different from that on POM (*p*=0.10). However, the *Streptococcus* spp. biofilm on Ultaire® AKP was significantly more homogeneous and structurally less differentiated when compared with that on the other test dental materials. Further investigation is warranted to determine the mechanism as to how Ultaire® AKP affects the structural maturation and composition of the *Streptococcus* spp. biofilm. While our study examined single- and dual-species environments for biofilm growth, multispecies models are being considered for future experiments.

Oral bacteria have been shown to colonize on and adhere to denture materials used for RPD*. In vitro* studies have demonstrated that *S. mutans, S. sanguinis,* and *C. albicans* adhere to POM [29], PMMA [30–33], and CoCr [30, 32]. Innate and acquired characteristics of RPD materials, such as hydrophobicity and surface roughness, can affect attachment and colonization by oral biofilms. Surface abrasion due to dentifrice cleansing can increase microbial attachment [33, 34], and material coating grafts that improve scratch resistance have been shown to inhibit *S. mutans* biofilm growth [35]. Further studies are needed to fully evaluate the impact of material surface roughness as it relates to the growth of biofilm.

Because the prevention of biofilm adhesion is recognized as one of the most efficient ways of controlling biofilm-related infections [36], the use of Ultaire® AKP in RPD manufacturing may show clinical value, pending the results of future *in vivo* studies. In this *in vitro* study, Ultaire® AKP demonstrated significantly less *C. albicans* biofilm growth at 24 hours compared with *Streptococcus* spp. It has been reported that acids or enzymes produced by microbial biofilms may lead to irregularities, cracks, or degradation of denture materials [5, 37]. Further long-term studies on how biofilm affects Ultaire® AKP properties are warranted.

The confocal imaging results revealed differences in size, shape, and structural organization of the 24-hour *Streptococcus* spp. biofilms on different test materials. The confocal image analysis of biofilm on Ultaire® AKP showed a significantly low roughness coefficient (a measurement of how the thickness of the biofilm varies, used to indicate biofilm heterogeneity) [28]. Because mature biofilms show structural variability in microbial subpopulations and extracellular matrices [38–40], these results suggest that 24-hour *Streptococcus* spp. biofilms on Ultaire® AKP were statistically more homogeneous and structurally less differentiated than those grown on the other test materials. Biofilms of undifferentiated cell populations and those of low structural complexity were found to be more susceptible to antimicrobial and cleaning agents [41]. Further *in vitro* studies are warranted to examine whether established biofilms on Ultaire® AKP are more vulnerable to cleansing agents than those grown on traditional dental materials. This could be of clinical relevance, since residual biofilms from current cleansing regimens may facilitate further regrowth of the biofilm [42].

In the current study, a static attachment assay and the CDC Biofilm Reactor were used to simulate the complex *in vivo* environment with sufficient simplification to achieve reproducible and statistically significant results. While other custom-built biofilm growth reactors are available to mimic the oral environment [43–45], the CDC Biofilm Reactor was chosen because it is standardized for regulatory claims [46, 47], is commercially available, and has previously been used in dental biofilm studies [26, 37, 48–50]. Other methods for simulation of the *in vivo* environment include *in situ* studies, which can be lengthy and uncomfortable for patients, and the use of donor-supplied biofilm populations is difficult to standardize [26].

The clinical impact of our study is that Ultaire® AKP demonstrated less *C. albicans* and *S. mutans* attachment than CoCr at 6 hours, which is an average timeframe for routine hygienic RPD cleansing. However, Ultaire® AKP also demonstrated reduced *C. albicans* biofilm growth and structurally less mature *Streptococcus* spp. biofilm at the 24-hour time point. This may be clinically relevant for elderly or disabled patients who are not able to maintain good oral hygiene regimens. Although this study demonstrated reduced short- and long-term biofilm growth on Ultaire® AKP, further clinical studies are warranted to validate the clinical relevance.

## 5. Conclusions

Compared with CoCr, Ultaire® AKP promoted significantly less *in vitro* attachment and biofilm growth of *C. albicans*. While Ultaire® AKP also promoted less cariogenic *Streptococci* attachment, comparable biofilm growth was noted but with less structural maturation. These properties—along with its flexibility, resistance to cleaner-induced surface roughness, and high impact strength and heat resistance similar to those of CoCr—make Ultaire® AKP an attractive option for use as a denture material in RPDs.

## Figures and Tables

**Table 1:** Attachment and biofilm growth of *Candida albicans* and *Streptococcus* spp. on selected dental materials.

	Test material	CFUs/coupon (mean ± SD)	% reduction^∗^	CFUs/coupon (mean ± SD)	% reduction^∗^
		*Candida albicans*		*Streptococcus mutans*	
6-hour static attachment	CoCr	2.4 ± 0.21 × 10^4^	0	3.2 ± 4.00 × 10^5^	0
	Ultaire® AKP	3.7 ± 0.33 × 10^3^	85^*∗∗*^	7.2 ± 4.70 × 10^4^	77^*∗∗*^
	PMMA	7.9 ± 0.65 × 10^3^	68^*∗∗*^	5.0 ± 1.30 × 10^4^	84^*∗∗*^

		*Candida albicans*		*Streptococcus* spp.	
24-hour dynamic biofilm growth	CoCr	4.3 ± 1.50 × 10^6^	0	3.0 ± 0.68 × 10^5^	75^*∗∗*^
	Ultaire® AKP	2.2 ± 0.50 × 10^6^	48^*∗∗*^	4.6 ± 0.95 × 10^5^	62
	PMMA	1.8 ± 0.63 × 10^6^	58^*∗∗*^	4.4 ± 0.90 × 10^5^	63
	POM	2.4 ± 1.20 × 10^6^	43	1.2 ± 2.20 × 10^6^	0

The procedures for the 6-hour attachment and 24-hour biofilm formation were as described in “Materials and Methods.” Values represent viable colony-forming units (CFU) of *C. albicans* and *Streptococcus* spp. on test material coupons. ^*∗*^% reduction = 100 × (CFU_CoCr or POM_-CFU_test coupon_)/CFU_CoCr or POM_. The viable bacterial CFUs (attachment or growth) of each test material was compared to each other material separately. For the reductions identified as statistically significant (^*∗∗*^*p* ≤ 0.05), the referent material is the one with the highest bacterial attachment or biofilm growth (“0” reduction).

## Data Availability

Data may be provided upon request to the corresponding author.
